# Comments on "Dissolution Enhancement of Atorvastatin Calcium by Cocrystallization"

**DOI:** 10.34172/apb.2021.067

**Published:** 2020-05-26

**Authors:** Shahram Emami, Ali Shayanfar

**Affiliations:** ^1^School of Pharmacy, Urmia University of Medical Sciences, Urmia, Iran.; ^2^Pharmaceutical Analysis Research Center and Faculty of Pharmacy, Tabriz University of Medical Sciences, Tabriz, Iran.

In a recent paper published in *Advanced Pharmaceutical Bulletin* by Al-Kazemi and colleagues,^1^ preparation of atorvastatin calcium trihydrate (ATC) cocrystals with nicotinamide and glucosamine have been reported and the authors declared that cocrystallization significantly improve the solubility and dissolution rate of the drug, in comparison to untreated ATC. There are certain problems with data and discussions presented in this paper, which give us cause to pen this commentary.

Before explaining our concerns about the work of Al-Kazemi and colleagues, we should consider some general information about cocrystals. Cocrystals are crystalline materials composed of two or more different molecular and/or ionic compounds generally in a stoichiometric ratio.^2,3^ In a routine study to investigate cocrystal formation between a drug and coformer, first, different stoichiometric ratios of components and various synthesis methods are attempted to find the right ratio and appropriate method of preparation. In the next step, the purity of new solid phases (cocrystal) is determined by using solid state analytical techniques such as powder X-ray diffraction (PXRD) and differential scanning calorimetry. Finally, physicochemical properties of the formed cocrystals are compared with the parent drug.^4,5^

Al-Kazemi et al have prepared the cocrystals in various stoichiometric ratios of ATC and glucosamine or nicotinamide and the cocrystals with the better physicochemical properties i.e. dissolution rate have been applied for characterization.^1^ Glucosamine is chemically unstable^6,7^ and is formulated as salt forms. By considering the melting point reported by the authors (differential scanning calorimetry data) for glucosamine and comparing the data with what reported in the literature,^8^ it seems that glucosamine hydrochloride, but not glucosamine, has been used in this study.

Although stoichiometric diversity has been reported for some cocrystals, cocrystal formation between a drug and particular coformer usually occurs only in a specific molar ratio. Most of the reported cocrystals in the literature have drug: coformer ratios of 1:1, 2:1, and 1:2.^9^ There are rare reports in the literature other than mentioned ratios.^10^ The authors examined 1:1, 1:3, and 1:10 ratios to prepare cocrystals. They do not provide any meaningful reason for choosing these ratios. The ratios of 1:3 and 1:10 are unusual and irrational for being investigated in cocrystal screening studies. In addition, each molecule of ATC contains two molecule of atorvastatin calcium which additionally complicates the process of selecting ratios.

Differential scanning calorimetry (DSC) is a powerful technique to characterize cocrystals. The thermal behavior of a cocrystal differs with those for the drug and coformer. Formation of a cocrystal is verified by the appearance of a new melting peak and disappearance of melting peaks of the drug and coformers in the DSC thermogram of the cocrystal.^11^ In Al-Kazemi and colleagues study, cocrystals did not demonstrate single melting peaks and their DSC data cannot support the formation of pure crystalline phases.

During saturation solubility studies, the reminded solid phase after solubility studies has been not characterized. A slight excess coformer exists as impurity in the structure of a cocrystal and full or partially instability of cocrystal in solution; cause the dependence of solubility on its components (drug and conformer) in the solution phase. In this state, the mass of excess solid phase is an important issue that should be considered.^12,13^

ATC after dissolving in a solvent and solvent evaporation is converted to a semi-crystalline phase.^14^ Therefore, a different PXRD pattern and decrease in crystallinity for the cocrystals from those of the individual components could be related to transformation of ATC crystalline form in the solvent evaporation process of cocrystal preparation.

Reported mass spectrum of ATC (Supplementary Figure 5) is not in agreement with reported spectrum in the literature^15^ and its molecular weight. In the Fourier transform infrared (FTIR) spectroscopy section, the authors compared FTIR spectrum of ATC with spectra of claimed cocrystals but surprisingly they ignored the role of water in the FTIR spectrum of ATC. We cannot agree with these comparisons. ATC molecule, in addition to atorvastatin calcium, contains water in its crystalline structure. Unfortunately, the authors did not provide any data about the presence or absence of water in the structure of their cocrystals. Without access to this data, it may not be possible to relate the shifts in the position of peaks to the cocrystal formation.

Finally, when we look at the scanning electron microscopy results, it can be seen in Figure 8, that authors specified drug and coformers particles on cocrystals. They also discussed in the text that: “The drug particles inside the cocrystal were transformed to smaller crystalline structures, which were finely dispersed and attached to the surface of the coformers particles”. These discussions about Figure 8 seem to be not correct. When a drug and coformer forms a cocrystal, a new crystalline phase is formed. Therefore, there are no separate drug or coformer particles. A cocrystal solid is a homogenous phase at molecular level and one cannot label a portion of the cocrystalline solid as coformer and another portion as drug particles.^16^

## Ethical Issues

Not applicable.

## Conflict of Interest

The authors declare that they have no conflict of interest.
